# Supplementary material for: 3D Chromatin Organization Involving MEIS1 Factor in the cis-Regulatory Landscape of GJB2
Source: Int J Mol Sci. 2022 Jun 23;23(13):6964. doi: 10.3390/ijms23136964 (PMC9266880; doi:10.3390/ijms23136964)
Supplement: Supplementary file 1 [file ijms-23-06964-s001.zip › ijms-1753977-supplementary.pdf]

Supplementary figure and tables

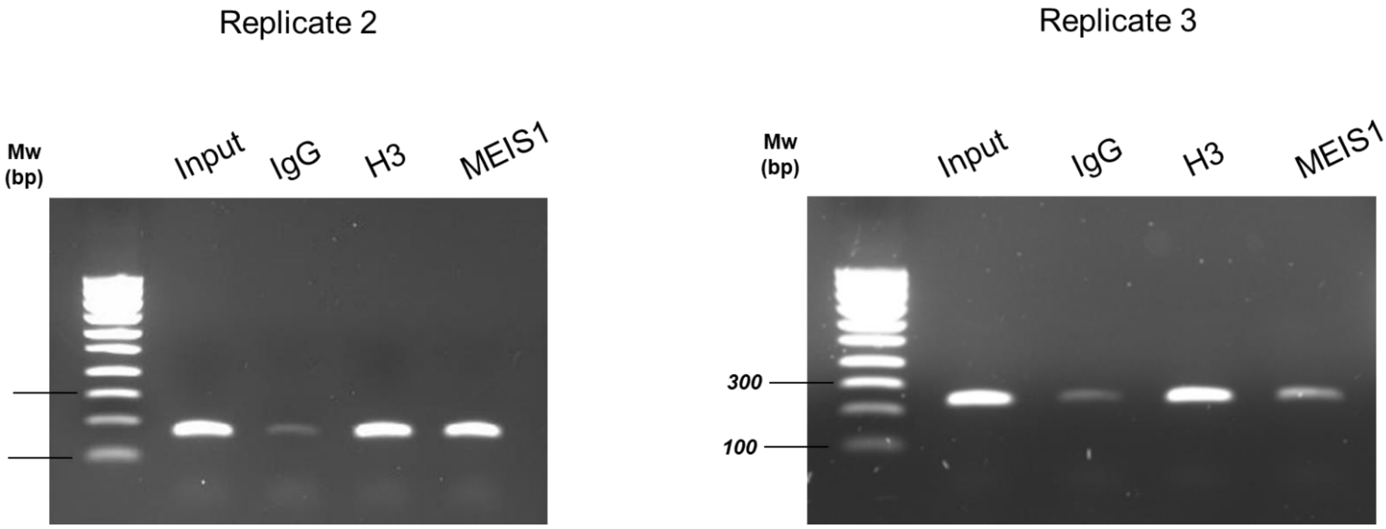

Figure S1. MEIS1 transcription factor contributes to *GJB2* expression, related to Figure 2.C

Binding of MEIS1 on C3 enhancer by ChIP-PCR. Two other replicates and in total three independent chromatin immunoprecipitations.

sgRNA#	target region	Sequence	PAM	Strand
1	P*GJB2*	GAGACTGGGAAAGTTACGGAG	GGG	+
2	C3	GCCTGTCAAGAGTCTTCCAAG	AGG	+
3	C3	GCTGCCCCACAATTACTCAC	AGG	+
4	C3	GTTGCTTCCCCCACATGCAAC	AGG	+
5	C3	AGCTTGTAAGTGCCGGCAAC	AGG	-

Table S1. CRISPRi and CRISPRa information, related to Figure 1.
Lists of sgRNA used for the both techniques.
G' was added because it is slightly more efficient

		Name of peaks	**genomic position chr13 (hg19)**			**distance from the *GJB2* transcriptional start site (kb)**		**length of interacting region (kb)**
	TSS GJB2		chr13	20 771 695				
	PGJB2		chr13	20 766 541	20 768 081			
<u>4C significant peaks</u>	*Viewpoint PGJB2*	-220	chr13	20 980 139	21 005 586	-208,4	-233,9	25,4
		-290	chr13	21 043 822	21 076 849	-272,1	-305,2	33
		-625	chr13	21 384 908	21 408 432	-613,2	-636,7	23,5
	Viewpoint C3	P	chr13	20 765 648	20 776 998	6	-5,3	11,3
		-20	chr13	20 786 728	20 796 959	-15	-25	10,2
		-625	chr13	21 387 057	21 400 524	-615,4	-628,8	13,5

Table S2. 4C peak coordinates, related to Figure 3 and 5.
PeakC calling determines genomic coordinates for each significant interactions.

Viewpoint	Direction	Orientation	Adaptater	Specific primer	Orientation	Primer sequence
P*GJB2*	Reading	5'	TACACGACGCTCTTCCGATCT	CTCCGCGACTCGAACAGATC	3'	TACACGACGCTCTTCCGATCTCTCCGCGACTCGAACAGATC
P*GJB2*	Non-reading	5'	ACTGGAGTTCAGACGTGTGCTCTTCCGATCT	GAGACAAGCCTCGTTTTTGCC	3'	ACTGGAGTTCAGACGTGTGCTCTTCCGATCTGAGACAAGCCTCGTTTTTGCC
C3	Reading	5'	TACACGACGCTCTTCCGATCT	GGAACCACTGGGTCAGAT	3'	TACACGACGCTCTTCCGATCTGGAACCACTGGGTCAGAT
C3	Non-reading	5'	ACTGGAGTTCAGACGTGTGCTCTTCCGATCT	GAATAAAAACTTGAGTAGCATG	3'	ACTGGAGTTCAGACGTGTGCTCTTCCGATCTGAATAAAAACTTGAGTAGCATG

Table S3. 4C-seq primers for PGJB2 and C3 viewpoints, related to Figure 3.

Lists of primers used for 4C-seq PCR.

cCRE	Direction	Orientation	Specific primer
P*GJB2*	Forward	5'	CGGTGAATTTAAAACGTTTGGTGGC
P*GJB2*	Reverse	5'	CCGCAGAATCCTATCAGTTTCC
-625-L	Forward	5'	CTTTAGGAGAGCTCTCAGTCAG
-625-L	Reverse	5'	GATCTTTCGCCTGTCTCCATAG
-625-R	Forward	5'	GATACTGCCACAGTAACTGAGG
-625-R	Reverse	5'	GGATCACACATCTGCTAGCTAC

Table S4. PCR primer sequences used for cloning into the luciferase reporter construct (5' - 3'), related to Figure 4.
